# Correction: Sarcopenia as a predictor of survival in patients undergoing bland transarterial embolization for unresectable hepatocellular carcinoma

**DOI:** 10.1371/journal.pone.0241715

**Published:** 2020-10-28

**Authors:** Ezio Lanza, Chiara Masetti, Gaia Messana, Riccardo Muglia, Nicola Pugliese, Roberto Ceriani, Ana Lleo de Nalda, Lorenza Rimassa, Guido Torzilli, Dario Poretti, Felice D’Antuono, Letterio Salvatore Politi, Vittorio Pedicini, Alessio Aghemo, on behalf of the Humanitas HCC Multidisciplinary Group

There are errors in the author affiliations. The correct affiliations are as follows:

Ezio Lanza^1^, Chiara Masetti^2^, Gaia Messana^3^, Riccardo Muglia^1,3^, Nicola Pugliese^2,3^, Roberto Ceriani^2^, Ana Lleo de Nalda^2,3^, Lorenza Rimassa^3,4^, Guido Torzilli^5,6^, Dario Poretti^1^, Felice D’Antuono^1^, Letterio Salvatore Politi^3,4^, Vittorio Pedicini^1^, Alessio Aghemo^2,3^

**1** Humanitas Clinical and Research Center–IRCCS—Division of Interventional Radiology, Department of Radiology, via Manzoni 56, 20089 Rozzano (Mi)–Italy **2** Humanitas Clinical and Research Center–IRCCS—Division of Internal Medicine and Hepatology, Department of Gastroenterology, via Manzoni 56, 20089 Rozzano (Mi)–Italy **3** Humanitas University, Department of Biomedical Sciences, Via Rita Levi Montalcini 4, 20090 Pieve Emanuele–Milan, Italy **4** Humanitas Clinical and Research Center–IRCCS—Medical Oncology and Hematology Unit, Humanitas Cancer Center, via Manzoni 56, 20089 Rozzano (Mi)–Italy **5** Humanitas Clinical and Research Center–IRCCS—Division of Hepatobiliary & General Surgery, Department of Surgery, via Manzoni 56, 20089 Rozzano (Mi)–Italy **6** Humanitas Clinical and Research Center–IRCCS—Department of Radiology via Manzoni 56, 20089 Rozzano (Mi)–Italy
