# Supplementary material for: Sarcopenia as a predictor of survival in patients undergoing bland transarterial embolization for unresectable hepatocellular carcinoma
Source: PLoS One. 2020 Jun 17;15(6):e0232371. doi: 10.1371/journal.pone.0232371 (PMC7299358; doi:10.1371/journal.pone.0232371)
Supplement: Attachment — Submitted filename: Response to Reviewers.docx [file pone.0232371.s002.docx]

**Response to Reviewers**

Dear Professor Alpini,

Thank you for giving us the opportunity to submit a revised draft of the manuscript “Sarcopenia as a predictor of survival in patients undergoing bland transarterial embolization for unresectable hepatocellular carcinoma”. We appreciate the constructive suggestions and the attentive comments you and the reviewers have made. We have incorporated most of them; changes are marked-up within the file “Revised Manuscript with Track Changes”.

Our response follows (Editor’s and reviewers’ comments are in *italics*).

**Journal requirements**

*1. Please ensure that your manuscript meets PLOS ONE's style requirements, including those for file naming. The PLOS ONE style templates can be found at* [*http://www.journals.plos.org/plosone/s/file?id=wjVg/PLOSOne_formatting_sample_main_body.pdf*](http://www.journals.plos.org/plosone/s/file?id=wjVg/PLOSOne_formatting_sample_main_body.pdf) [*http://www.journals.plos.org/plosone/s/file?id=ba62/PLOSOne_formatting_sample_title_authors_affiliations.pdf*](http://www.journals.plos.org/plosone/s/file?id=ba62/PLOSOne_formatting_sample_title_authors_affiliations.pdf)

**Reply**: The corrections have been made.

*2. Please amend your current ethics statement to address the following concerns: Please state whether informed consent was written or verbal. If written consent was not obtained, please describe how you recorded/documented participant consent, and if the ethics committees/IRBs approved this consent procedure.*

**Reply**: We specified in the text that informed consent was written. Thank you for pointing this out.

*3. One of the noted authors is a group or consortium "Humanitas HCC Multidisciplinary Group" In addition to naming the author group, please list the individual authors and affiliations within this group in the acknowledgements section of your manuscript. Please also indicate clearly a lead author for this group along with a contact email address.*

**Reply**: We added the members list of Humanitas HCC Multidisciplinary Group in the Acknowledgments section.

*4. Please include captions for your Supporting Information files at the end of your manuscript, and update any in-text citations to match accordingly. Please see our Supporting Information guidelines for more information:* [*http://journals.plos.org/plosone/s/supporting-information*](http://journals.plos.org/plosone/s/supporting-information)*.*

**Reply**: We added captions for our Supporting Information.

**Review Comments to the Author**

***Reviewer #1****: This manuscript is a mono-centric study by the Humanitas HCC Multidisciplinary Group in Milan (Italy). It is a retrospective study aiming to evaluate the prognostic significance of sarcopenia in patients affected by HCC who received bland transarterial embolization (TAE) therapy. The manuscript is interesting and well-written. I have only few minor points.*

*- In table 1, authors could include also alcohol abuse and NAFLD diagnosis as variables.*

*- Line 179. Authors stated “No specific liver disease was associated with sarcopenia (Table 4); non-NASH liver disease showed the highest incidence (p < 0.01)”. However, table 4 reports variables tested in the multivariate analysis.*

**Reply**: We included in Table 1 the variables “Alcohol abuse” and “NASH” as suggested. We removed the reported sentence because there was an error, thank you for pointing this out.

***Reviewer #2****: The authors provide insight on an important factor determining HCC TAE outcome, however there are some issues that must be addressed prior to publication:*

*1. Ranges must be included along with the mean in the various tables provided with this manuscript. Additionally, subcategories of underlying diseases of sarcopenic and non-sarcopenic patients must be added in additional rows in the appropriate table.*

**Reply**: Ranges were included in the tables for continuous variables. HBV+, NASH and alcohol abuse were added as variables in Table 1.

*2. There must a table with the TAE data that was listed in the manuscript.*

**Reply**: We created a table with the TAE data, specifying the number and types of TAE procedures performed (Table 2).

*3. Results should be rewritten for a more cohesive presentation of the data collected.*

**Reply**: We reorganized the data included in the Results in three main paragraphs: “Descriptive data of the study population”, “Impact of sarcopenia on the safety of TAE”, “Impact of sarcopenia on survival”. We believe that details are necessary for the deep comprehension of the study and should be included in the text. Instead, to have a general schematic view, Tables should be used.

*4. Correlation analysis should be performed between sarcopenia and the following: a. Previous treatment and HCC nodule # b. TAE complication and TAE #*

**Reply**: We have tested the interactions and both proved non-significant (previous treatments#HCC, p = 0.070; TAE complication#number of TAE, p = 0.65). We have added these data in the “Materials and Methods” and “Results” section. The sentences read:

“We tested the following potential interactions: TAE-related complications and number of treatments; previous treatments and number of HCC nodules.” (Materials and methods)

“The potential interactions proved non-significant and were not included in the final model.” (Results)

*5. Additionally, there should be analysis that separates the OS of sarcopenic/non-sarcopenic patients for the various stages. This data will likely be weaker due to the lower N, but it will help lend evidence to the idea that sarcopenia may influence OS and early intervention will help depending on HCC stage.*

**Reply:** We generated Kaplan-Meier curves according to disease stage (BCLC), distinguishing sarcopenic patients from non-sarcopenic patients. They are incorporated in Fig 2.

*6. The authors should discuss the lower OS in non-sarcopenic patients in years 3 and 4 compared to sarcopenic patients.*

**Reply**: The lower OS at 4- and 5-years in the non-sarcopenic group with respect to the sarcopenic group was examined in the Discussion.
